# Author Correction: From bulk effective mass to 2D carrier mobility accurate prediction via adversarial transfer learning

**DOI:** 10.1038/s41467-024-50561-0

**Published:** 2024-07-31

**Authors:** Xinyu Chen, Shuaihua Lu, Qian Chen, Qionghua Zhou, Jinlan Wang

**Affiliations:** 1 Key Laboratory of Quantum Materials and Devices of Ministry of Education, School of Physics, Southeast University, Nanjing, China; 2 Suzhou Laboratory, Suzhou, China

**Keywords:** Two-dimensional materials, Computational methods

Correction to: *Nature Communications* 10.1038/s41467-024-49686-z, published online 25 June 2024

The original version of this Article contained an error in Figure 4a, in which the label of the final stage of the material screening process reporting their carrier mobility was incorrectly given as ‘μ < 10^4^ cm^2^V^−1^s^−1^’. The corrected label is ‘μ > 10^4^ cm^2^V^−1^s^−1^’. This has been corrected in both the PDF and HTML versions of the Article.

