# Supplementary material for: From bulk effective mass to 2D carrier mobility accurate prediction via adversarial transfer learning
Source: Nat Commun. 2024 Jun 25;15:5391. doi: 10.1038/s41467-024-49686-z (PMC11199574; doi:10.1038/s41467-024-49686-z)
Supplement: Supplementary file 1 — Supplementary Information [file 41467_2024_49686_MOESM1_ESM.pdf]

Supplementary Information: From bulk effective mass to 2D carrier mobility accurate prediction via adversarial transfer learning

Xinyu Chen[1], *Shuaihua Lu*[1], *Qian Chen*[1], *Qionghua Zhou*[1,2,*] and *Jinlan Wang*[1,2,*]

[1]Key Laboratory of Quantum Materials and Devices of Ministry of Education, School of Physics, Southeast University, Nanjing 21189, China

[2] Suzhou Laboratory, Suzhou, China

[*]Corresponding authors: Jinlan Wang (jlwang@seu.edu.cn) and Qionghua Zhou (qh.zhou@seu.edu.cn)

Table of contents

1. Data distribution....2
2. Model optimization and comparison....4
3. Model interpretation....8
4. High-throughput carrier mobility calculation. 10

1. Data distribution.

The completeness of the dataset is crucial for ensuring the reliability and generalizability of machine learning (ML) models. In order to train an accurate and robust model, it is important to have a balanced dataset that covers a large element and property space. In this study, the training and testing datasets were collected from published papers based on theoretical calculations using the deformation potential approximation method, resulting in a total of 178 samples. Prediction set are two open-source 2D material databases, C2DB[1] and 2Dmatpedia[2]. The element and property distribution of different data sets are shown in Supplementary Fig. 1 and Supplementary Fig. 2. As shown in Supplementary Fig. 1, the training and testing datasets cover a large element space and the average carrier mobilities follow a normal distribution, which facilitates effective model training.

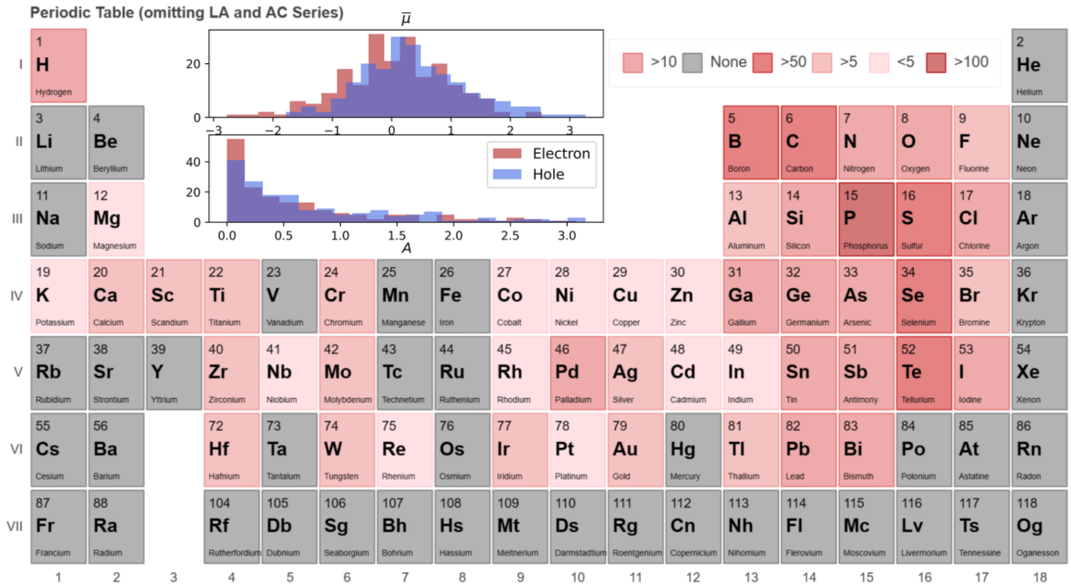

Supplementary Figure 1. Element distribution of training and testing data. The insert histogram shows the distribution of average carrier mobility and mobility anisotropy.

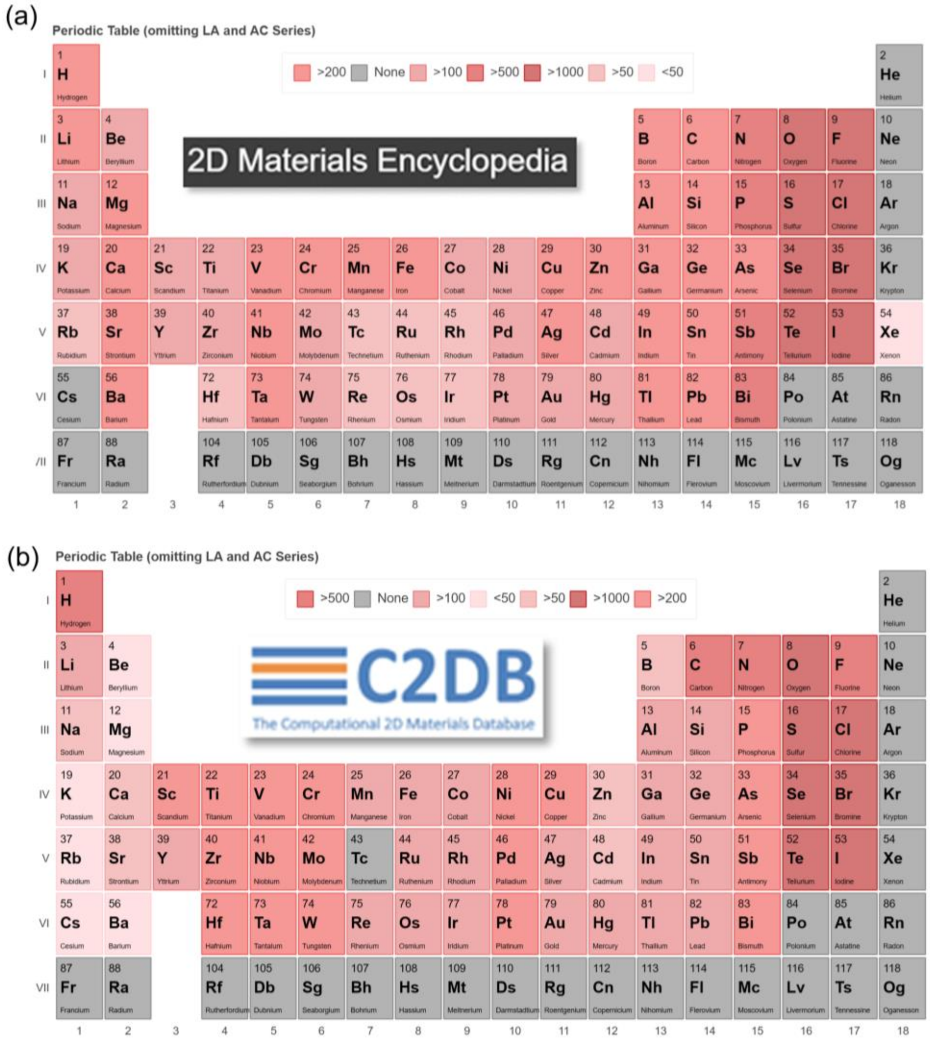

Supplementary Figure 2. Element distribution of prediction sets. Which are (a) 2Dmatpedia and (b) C2DB.

2. Model optimization and comparison.

The multi-layer perceptron (MLP) model is used to build adversarial transfer-learning network structure, which is a fully-connected neural network with multiple layers. We further optimize the number of network layers and the neurons in each layer by random search. The joint loss is the summation of the regressor loss (MAELoss) and the reversed classifier loss (BCELoss), which are shown in Supplementary Fig. 3(a-b). An intriguing observation is that deeper hidden layer result in better feature extraction. This trend can be attributed to two factors. Firstly, the regulation of adversarial training improves the effectiveness of the network in the target domain. Additionally, there is a close relationship between effective mass and carrier mobility, which may contribute to this trend. Furthermore, the lower dimensionality of deeper layers may provide a bonus when working with small target datasets. It is worth noting that training an MLP-based classifier can be challenging when dealing with lower dimensionality data. In such cases, the use of simpler models such as support vector machines and logistic regression may help to improve training efficiency.

The optimized network structures are demonstrated in Supplementary Fig. 3(c-d), which are pyramid-like MLP, that have been proved to be effective for material property prediction in ElemNet[3] and it is easy to build. To further improve model performance, one can introduce multi-task learning and graph-based method.[4,5]

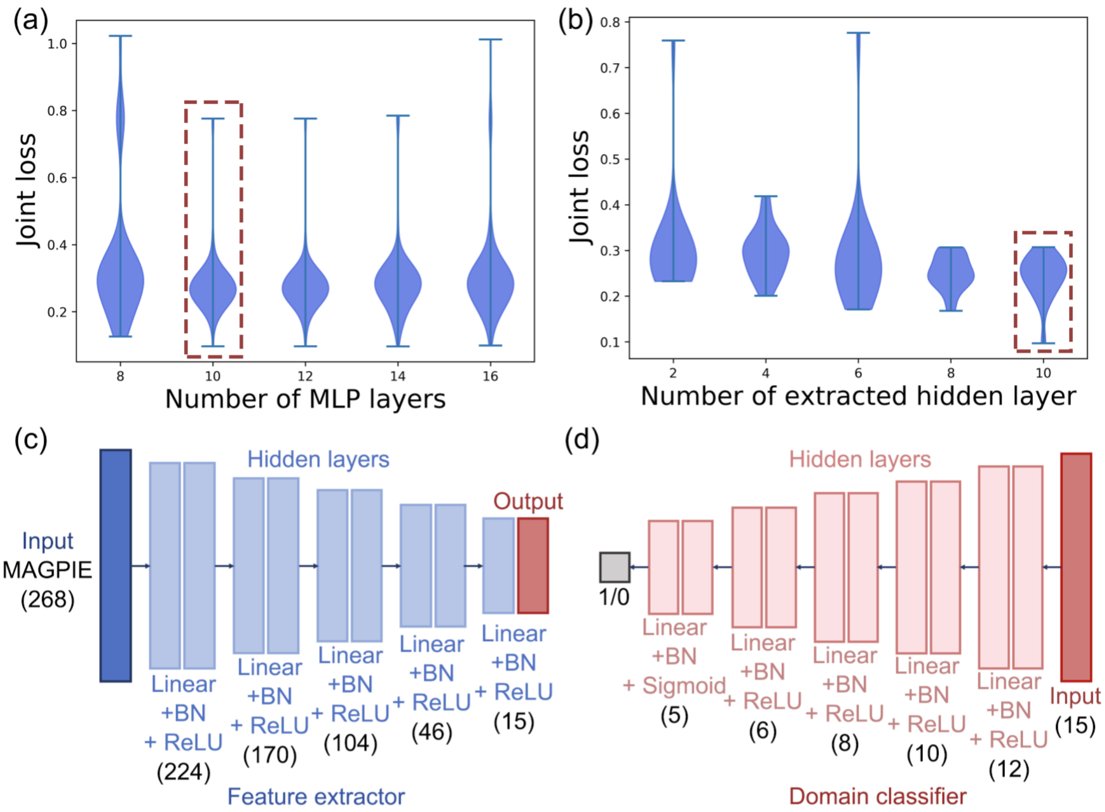

Supplementary Figure 3. Model optimization for adversarial transfer learning. Joint loss of MLP models with (a) different layers and (b) optimal layers with different neurons in each layer. The red dashed line box selects the optimal network configuration. The optimal (c) feature extractor and (d) data source classifier, each hidden layer contains a linear and a batch normalization (BN) layer with rectified linear unit (ReLU) as activation function, the output of classifier uses Sigmoid as activation function. The input feature is generated by materials agnostic platform for informatics and exploration (MAGPIE). Source data are provided as Source Data file.

The adversarial training workflow is illustrated in Supplementary Fig. 4. We begin by removing duplicated samples and outliers from the input source and target data. Next, we select a range of learning rates and use Adam optimizer to initialize the feature extractor multiple times. If the loss drops over 30% within 50 epochs, the MLP initialization is deemed successful. We then train the initialized MLP model for 500 epochs to capture knowledge in the source domain. The trained feature extractor produces two sets of features for the source and target data, respectively, which are utilized to train the classifier. The classifier is also initialized using this process.

We then fix the classifier and continue training the feature extractor. However, the loss function is adjusted by incorporating another term from the reversed classifier loss. Given the vast differences between the source and target domains, the initial classifier can easily identify the data sources. When the feature extractor becomes good enough to fool the classifier, we fix the feature extractor and train the classifier again. This process of training the feature extractor and classifier alternately is repeated several times until the classifier can no longer distinguish the origin of the data.

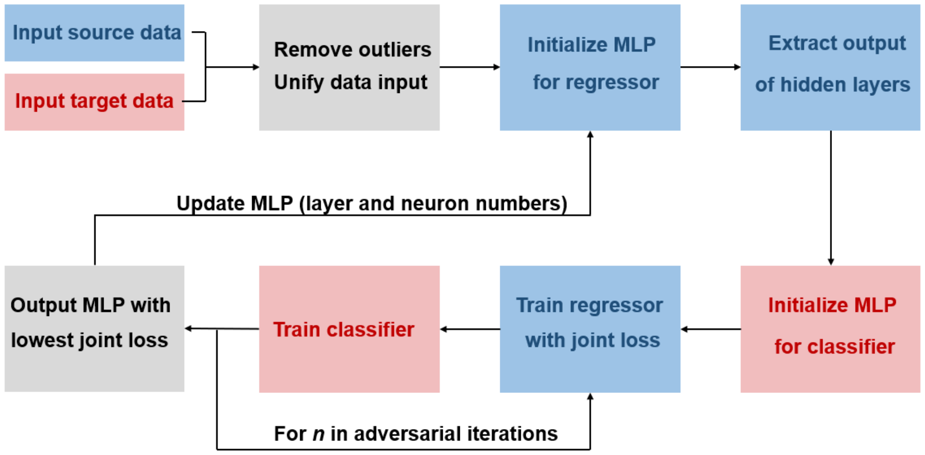

Supplementary Figure 4. Adversarial transfer-learning workflow. The arrows represent the order of data processing or model training.

The extracted features are used as input to train a gradient boosting tree model, which is more effective for small datasets. We also compared other machine learning models as shown in Supplementary Fig. 5(a-b), gradient boosting tree model under XGBoost framework gives highest R^2 score and lowest MAE. The hyperparameters of LASSO and KRR models are optimized by grid search, and for XGB models, we use a global search algorithm based on the simulated annealing algorithm[6] to find the optimal parameters, which are listed in Supplementary Table 1. To get better prediction accuracy, and fully use the calculated results, we using active learning to sample new materials with highest predicted carrier mobilities and add them back to update models, 44 samples are added back in 11 iterations, the performance increment are shown in Supplementary Fig. 5(c).

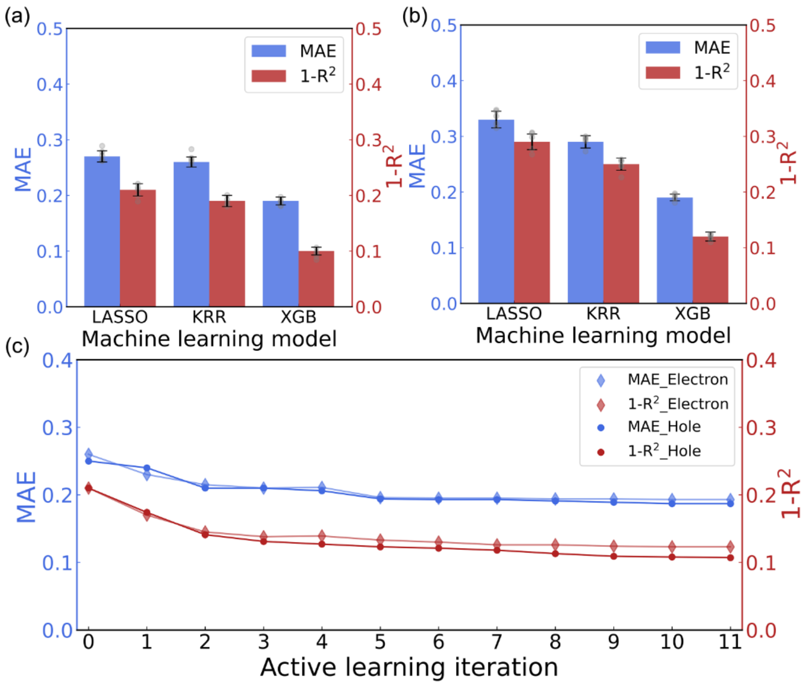

Supplementary Figure 5. Selection of mobility prediction models and active learning sampling. Model comparison on predicting (a) average electron mobility and (b) average hole mobility. The model performance is evaluated by mean absolute error (MAE) and coefficient of determination (R^2). Three popular models, least absolute shrinkage and selection operator (LASSO), kernel ridge regression (KRR), extreme gradient boosting (XGB) are chosen for comparison. All models are trained five times with different random seed to split data, the error bar represent the standard deviation. (c) Performance increment during active learning. The solid dots and transparent diamonds represent hole and electron, respectively. Source data are provided as Source Data file.

Supplementary Table 1 Hyperparameters' search range and the optimal values for different models.

range / models	colsample_ bytree (0.5-1)	max_ depth (1-10)	n_ estimator (10-200)	learning_ rate (0.01-0.5)	sub sample (0.5-1)	min_child_ weight (0.5-10)	Gamma (0.001-0.5)
$\overline{\mu}_{e-DPT}$	0.84	5.00	86	0.49	0.95	4.75	0.26
$\overline{\mu}_{h-DPT}$	0.84	5.00	112	0.44	0.96	4.66	0.23
A_e	0.70	6.00	116	0.47	0.95	5.56	0.41
A_h	0.65	5.00	93	0.33	0.97	5.30	0.38
$\overline{\mu}_{e-EPC}$	0.81	4.00	114	0.37	0.99	3.42	0.16
$\overline{\mu}_{h-EPC}$	0.80	5.00	107	0.35	0.95	4.40	0.24

3. Model interpretation.

The Shapley additive explanation (SHAP) value can reflect the positive or negative impact of each feature in each sample on the prediction results, providing deeper interpretation capabilities for complex ML models. Supplementary Fig. 6 shows the SHAP value of all interpretable features on predicting average mobility and mobility anisotropy. The descriptions of these features are listed in Supplementary Table 2.

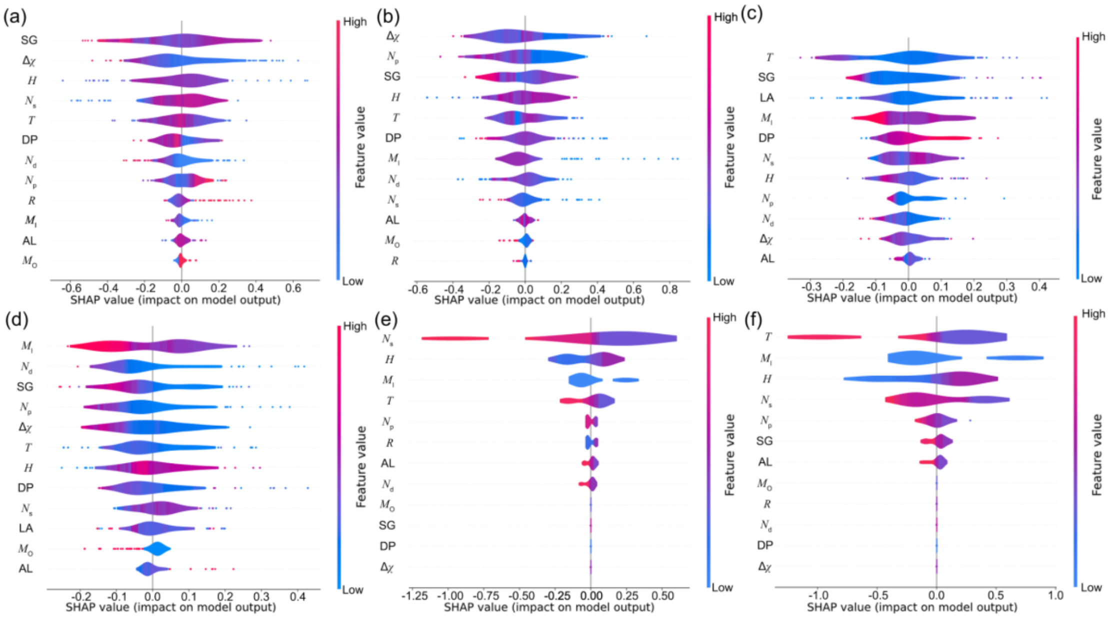

Supplementary Figure 6. Model interpretation with Shapley additive explanation (SHAP). Global interpretation into (a) average DPT electron mobility; (b) average DPT hole mobility; (c) electron mobility anisotropy; (d) hole mobility anisotropy (e) average EPC electron mobility; (f) average EPC hole mobility regression using SHAP value for each feature. Sorted in descending order of importance. Source data are provided as Source Data file.

Supplementary Table 2. Feature list of four feature groups. Feature label and the corresponding description, all features are divided into four sets according to the way they are extracted or their physical means, and the letters in brackets are their abbreviations.

Feature set	Symbol	Description
ATL features (ATL)	ATL1-15	15 outputs from the cross-domain adversarial transfer learning
Symmetry (SYM)	SG	Space group number
	R	Number of in-plane rotate symmetry operation
	M_I	Number of in-plane mirror symmetry operation
	M_O	Number of out-of-plane mirror symmetry operation
Geometry (GEO)	T	Thickness
	AL	Atom layers
	LA	Lattice anisotropy defined as the long lattice divide short one
	H	Buckling height defined as H = T/(AL-1)
Electron features (ELE)	$\Delta\chi$	Maximum Pauling electronegativity difference
	DP	Average dipole-moment
	$N_{s,p,d}$	Average fraction of valence electrons in the s,p, and d shells of the constituent elements

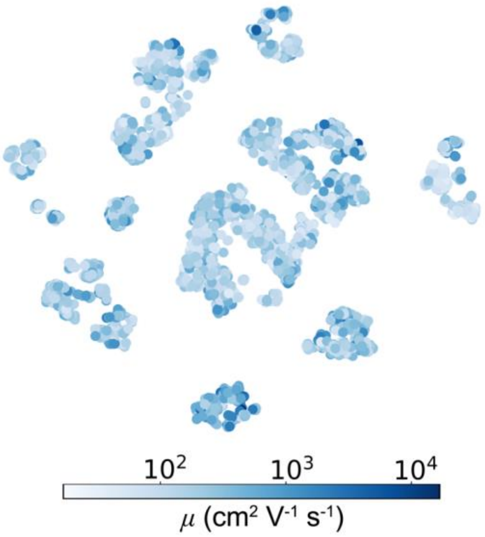

Supplementary Figure 7. Visualization of latent feature space by t-distributed stochastic neighbor embedding (t-SNE). The scatter plot uses color to indicate the hole mobility of 2D semiconductors. Source data are provided as Source Data file.

4. High-throughput carrier mobility calculation.

Supplementary Fig. 8 shows the overall calculation work flow of 2D carrier mobility, considering calculation efficiency, only materials with atom number less than 36 in the transformed orthogonal cell are calculated. The first-order and second-order fitting of deformation potential are automatically done using Scipy package, and the fitness (R^2) are calculated, only the results with fitness over 0.9 are presented. Supplementary Fig. 9 presents the predicted mobility versus calculated ones, the MAE for both electron and hole mobility are below 0.2, indicating our model gives accurate predictions. Supplementary Table 3 summarized some critical parameters of selected materials such as deformation potential and effective mass along *x* and *y* directions. Their crystal and electronic structures are given in Supplementary Fig 11-16.

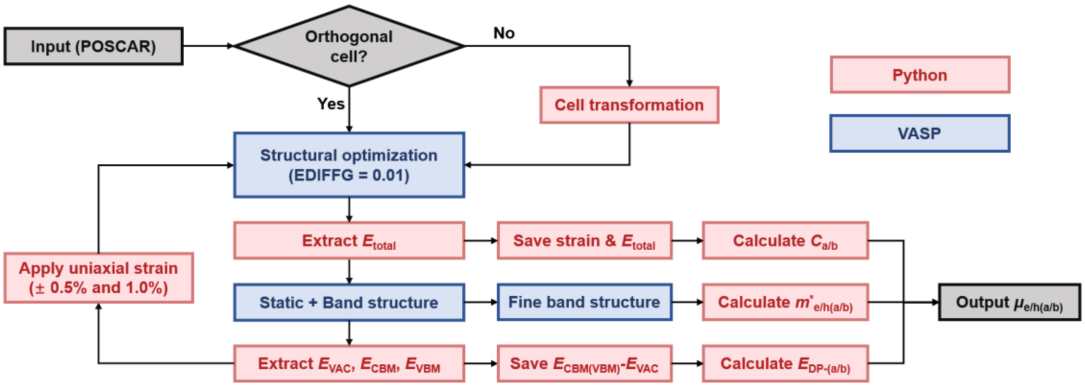

Supplementary Figure 8. Automatic carrier mobility calculation workflow. The red box are steps using python script and the blue box are steps calculated by VASP.

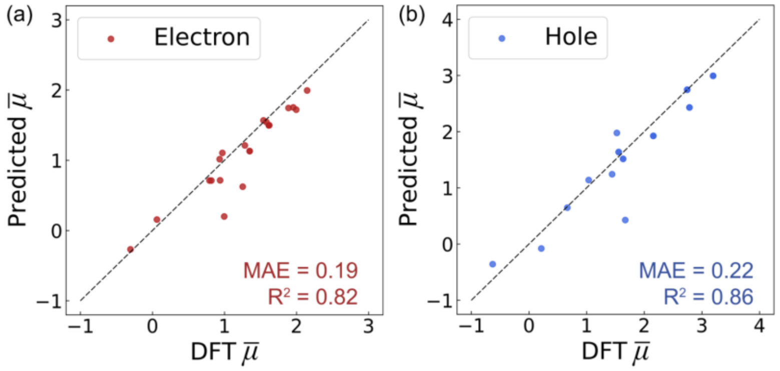

Supplementary Figure 9. Model evaluation on prediction sets. Prediction accuracy on predicting the (a) average electron mobility and (b) hole mobility. The model performance is assessed using mean absolute error (MAE) and coefficient of determination (R^2), with the corresponding values displayed as text. Source data are provided as Source Data file.

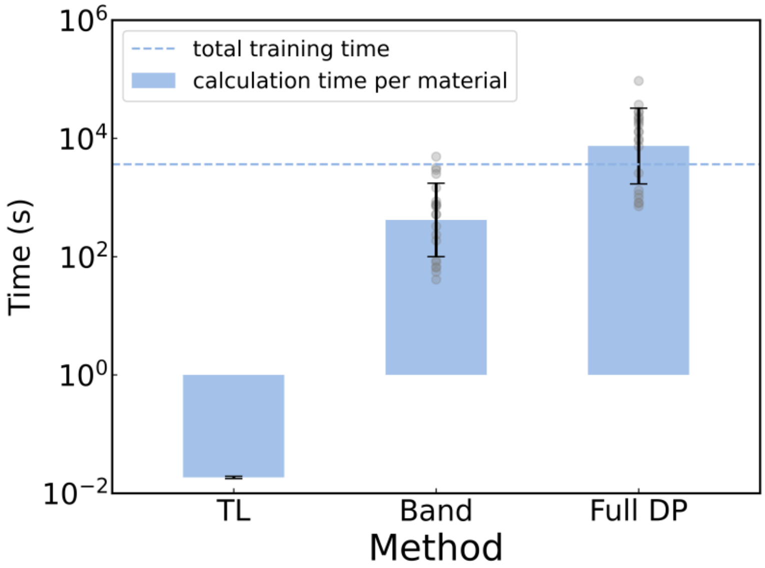

Supplementary Figure 10. Time cost comparison of different mobility evaluation methods. We compared the trained transfer learning model (TL), DFT-based band structure calculation (Band) and deformation potential calculation (Full DP), the error bar indicates the standard deviation on calculating different materials and the dashed line is the time cost to train TL model. TL predictions are performed on single core of Intel Xeon Gold 5218, TL model training is performed on Nvidia RTX 2080Ti and single core of Intel Xeon Gold 5218, and the DFT calculations are performed on 48 cores of Intel Xeon Gold 6248R. The data are presented as mean values +/- standard deviation of 21 samples. Source data are provided as Source Data file.

Supplementary Fig. 10 provides a comparison of the time costs associated with TL-based mobility prediction, DFT-based band structure calculation and deformation potential calculation. Our TL model demonstrated computational speed improvements of four to five orders of magnitude when compared to DFT calculations. Additionally, the overall training time for our model was found to be similar to the time required for calculating the deformation potential of a single material, and we don't need to retrain the model when perform predictions. It is worth noting that the time cost of DFT is strongly dependent on the cell size and atom number, resulting in considerable variation in computation times across different materials, which can be seen from the large error bar. In contrast, the network complexity of TL is fixed, which ensures that its prediction speed will not be significantly slowed down, regardless of the structural complexity of the material being predicted.

Supplementary Table 3. Comparison of running time and prediction accuracy, the running time are the estimated time for predicting 1000 2D materials, the prediction accuracy is evaluated by R^2 score compared of DFT-based calculations.

Method	Accuracy	Time (seconds)
DFT-deformation potential	1.00	~10^7
**Hybrid transfer learning (this work)**	**0.89**	**~2×10^3 (train) / ~10 (predict)**
Feature-based transfer learning	0.37	~5×10^2 (train) / ~10 (predict)
Scratch multilayer perceptron	0.55	~2×10^2 (train) / ~10 (predict)
LASSO	0.61	~10 (train) / ~10^{-1} (predict)
Kernel Rigid Regress	0.60	~10 (train) / ~10^{-1} (predict)

Supplementary Table 4. Calculated carrier mobility, effective mass and deformation potential for the top ten materials with the highest carrier mobility. All materials are converted to orthogonal lattices, which are labeled as *x* and *y*. Since the deformation potential approximation and effective mass approximation require first-order and second-order fitting, results with a fitness less than 0.9 may be unreliable thus are represented by '/'.

Material	Space group	E_g (eV)	carrier	E_x (eV)	E_y (eV)	m^*_x (m_0)	m^*_y (m_0)	μ_x (10^3 cm²/V·s)	μ_y
BiSb	$P2_1$	0.52	e	6.26	2.82	0.03	0.07	18.35	8.05
			h	8.23	1.91	0.04	0.07	17.81	31.70
BiAs	$P2_1$	0.55	e	6.81	1.82	0.05	0.08	10.09	53.96
			h	8.24	1.36	0.04	0.06	10.35	158.11
$GeBi_2$	$Pmc2_1$	0.57	e	5.53	0.24	0.07	0.31	3.36	131.88
			h	1.93	2.38	0.33	0.91	1.53	0.13
Sb_2OSe_2	P1	0.62	e	6.96	5.89	0.14	0.03	2.74	10.70
			h	3.45	3.74	0.73	0.41	0.27	0.27
$PbSe_3$	P1	0.74	e	0.55	1.95	0.19	0.73	23.48	0.22
			h	2.11	0.58	0.36	1.02	0.25	1.10
$GeSb_2$	$Pmc2_1$	0.78	e	6.76	0.64	0.14	0.33	0.88	11.35
			h	1.79	4.21	0.40	1.52	1.17	0.02
AgI	Pmma	0.85	e	1.64	1.08	0.29	0.30	4.64	9.47
			h	1.99	1.85	0.74	0.28	0.79	0.31
BiISe	Pmmn	1.10	e	9.04	1.63	0.09	0.52	0.86	3.91
			h	1.68	5.22	0.16	0.29	13.90	0.69
In_4Se_3	$Pmn2_1$	1.13	e	4.11	1.05	0.31	0.13	0.81	23.84

			h	0.72	4.16	2.48	0.13	1.16	0.54
AsS	$P2_1/c$	1.20	e	11.52	2.74	2.89	0.39	0.01	0.11
			h	0.32	5.32	0.45	0.13	61.91	0.37
PbTe	P4/nmm	1.26	e	1.14	1.14	0.14	0.14	32.78	32.78
			h	6.44	6.44	0.15	0.15	0.93	0.93
Ga_2Te_3	Pc	1.48	e	2.81	2.63	0.09	0.06	6.81	10.41
			h	4.89	5.66	0.65	0.45	0.05	0.05
$CuBrO_2$	$P222_1$	0.49	e	1.55	1.96	1.34	18.58	0.02	0.01
			h	2.19	0.56	0.67	0.37	0.21	27.43
Nb_2SiTe_4	Pbam	0.54	e	2.29	1.59	0.23	0.30	6.27	9.70
			h	0.68	2.80	2.43	36.41	0.19	0.01
SiF	$P\bar{3}m1$	0.66	e	1.45	1.28	0.17	0.40	11.85	5.78
			h	1.60	2.43	0.60	0.10	2.99	6.81
$SnSb_2$	$Pmc2_1$	0.69	e	7.38	0.59	0.11	0.31	0.90	12.43
			h	2.59	1.97	0.31	1.18	0.82	0.09
NiS_2	$P2_1$	0.80	e	6.67	6.51	1.25	0.33	0.03	0.20
			h	0.26	/	0.69	0.66	32.07	/
InP_5	$Pmc2_1$	1.20	e	1.66	0.18	0.41	1.29	2.54	22.31
			h	1.47	/	3.63	/	/	/
GeSe	Pbcm	0.49	e	0.44	4.11	0.26	0.08	107.96	2.31
			h	5.32	0.64	0.27	/	/	/
As_2Se_3	$P2_1/m$	0.72	e	11.51	2.01	0.08	0.13	1.70	11.86
			h	6.70	/	0.30	/	/	/
BiClSe	Pmmn	1.17	e	9.11	0.29	0.06	1.11	1.49	45.72
			h	2.53	1.49	0.55	/	/	/

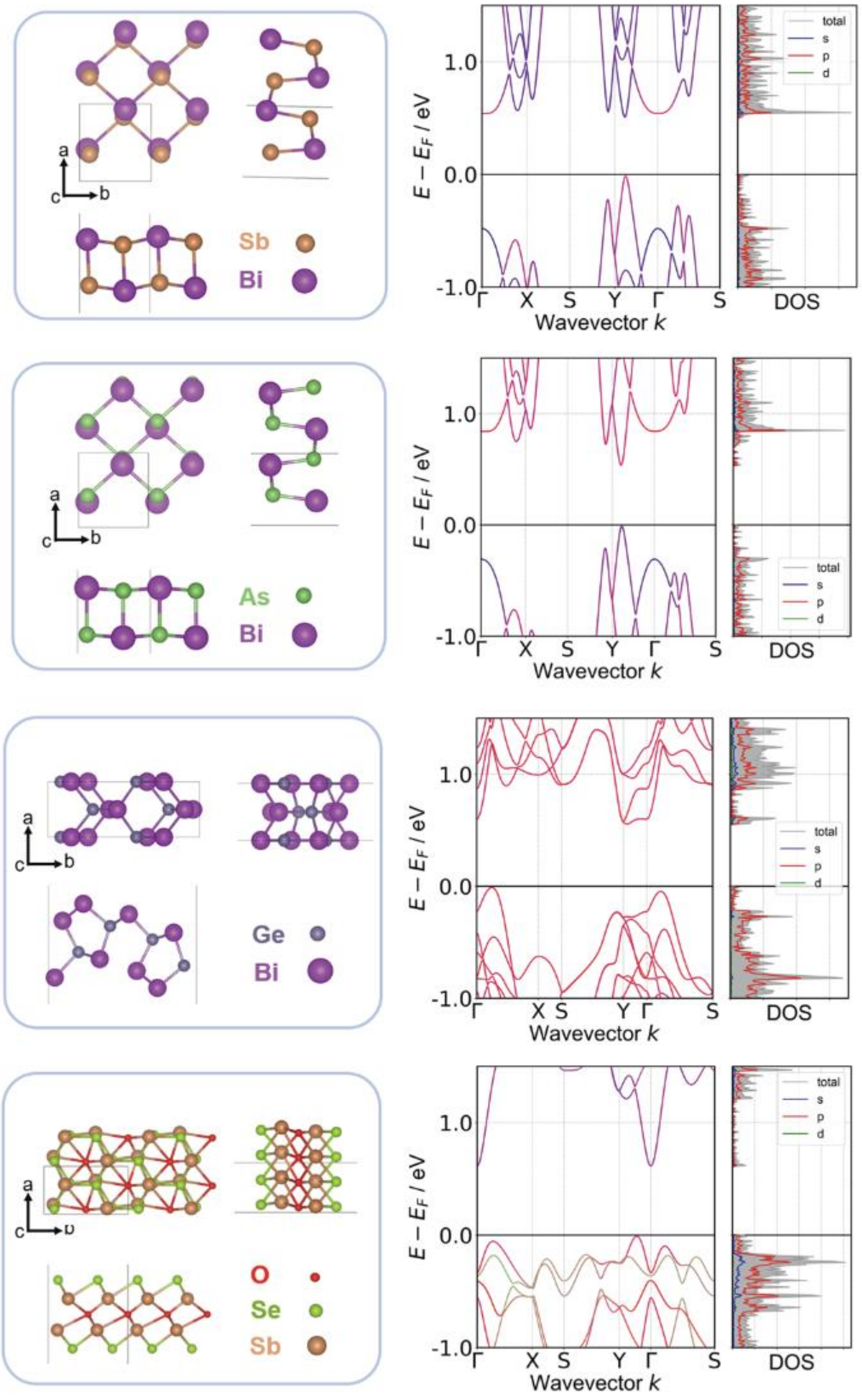

Supplementary Figure 11. Crystal and electronic structures of BiSb, BiAs, $GeBi_2$ and Sb_2OSe_2.

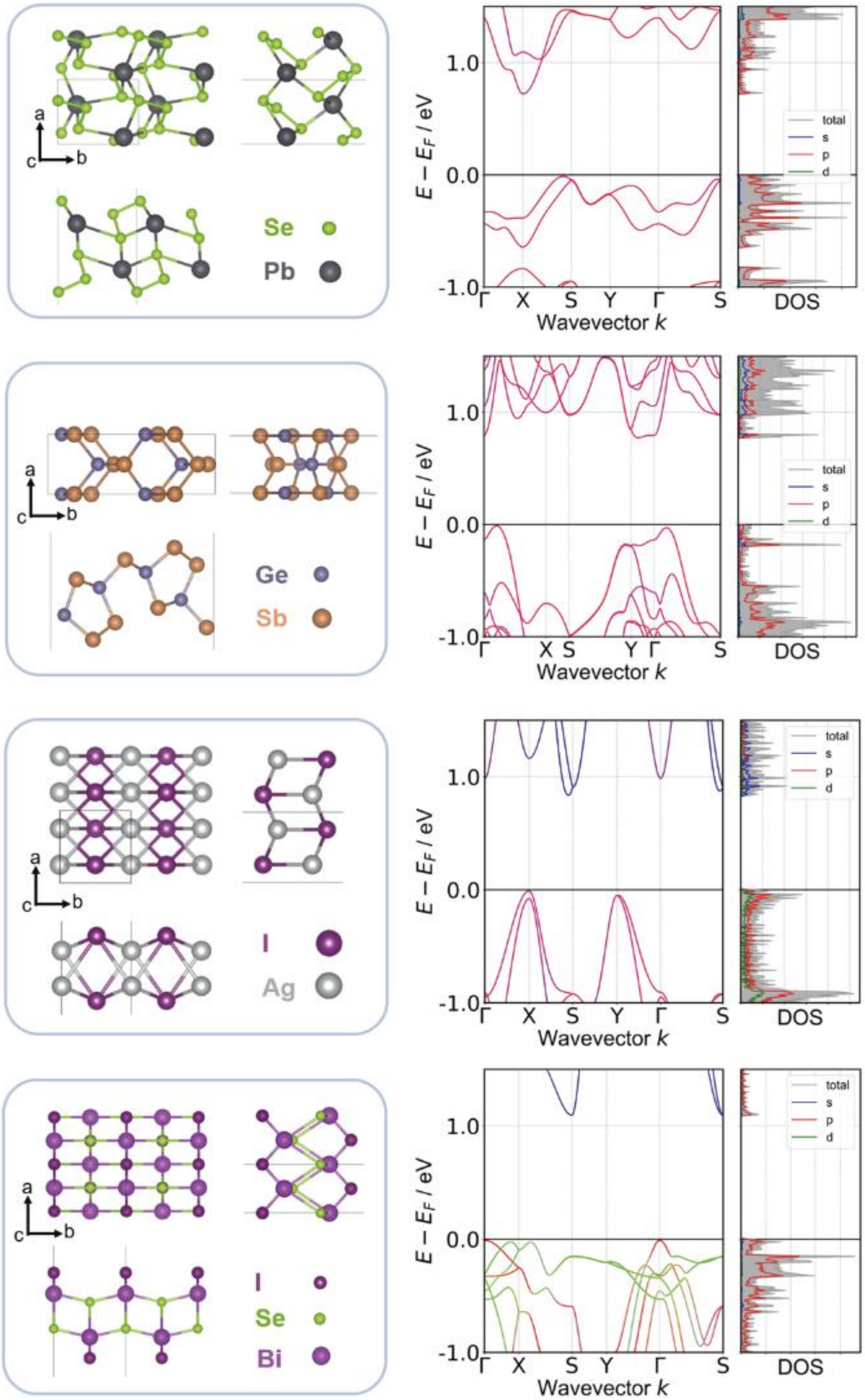

Supplementary Figure 12. Crystal and electronic structures of $PbSe_3$, $GeSb_2$, AgI and BiISe.

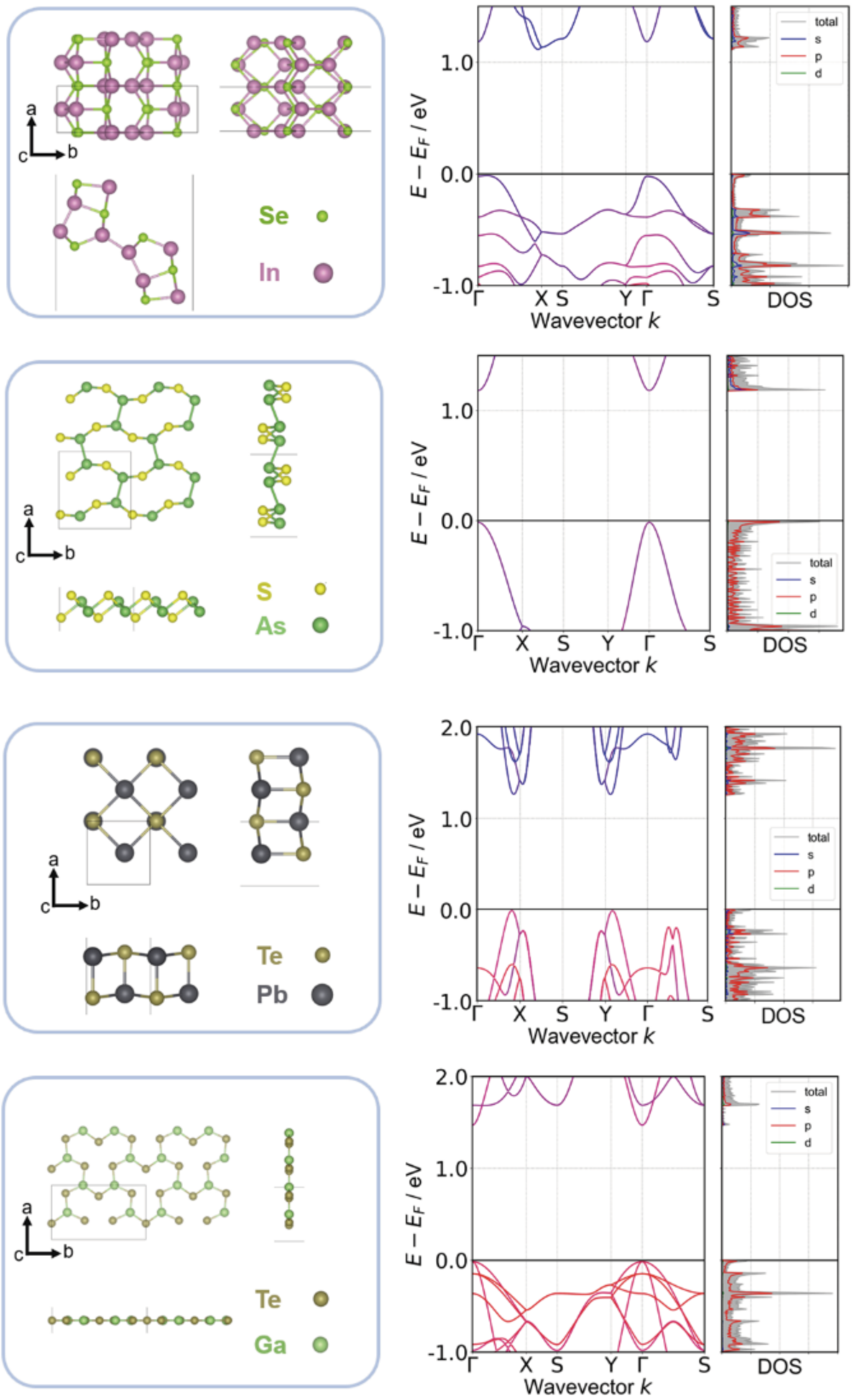

Supplementary Figure 13. Crystal and electronic structures of In_4Se_3, AsS, PbTe and Ga_2Te_3.

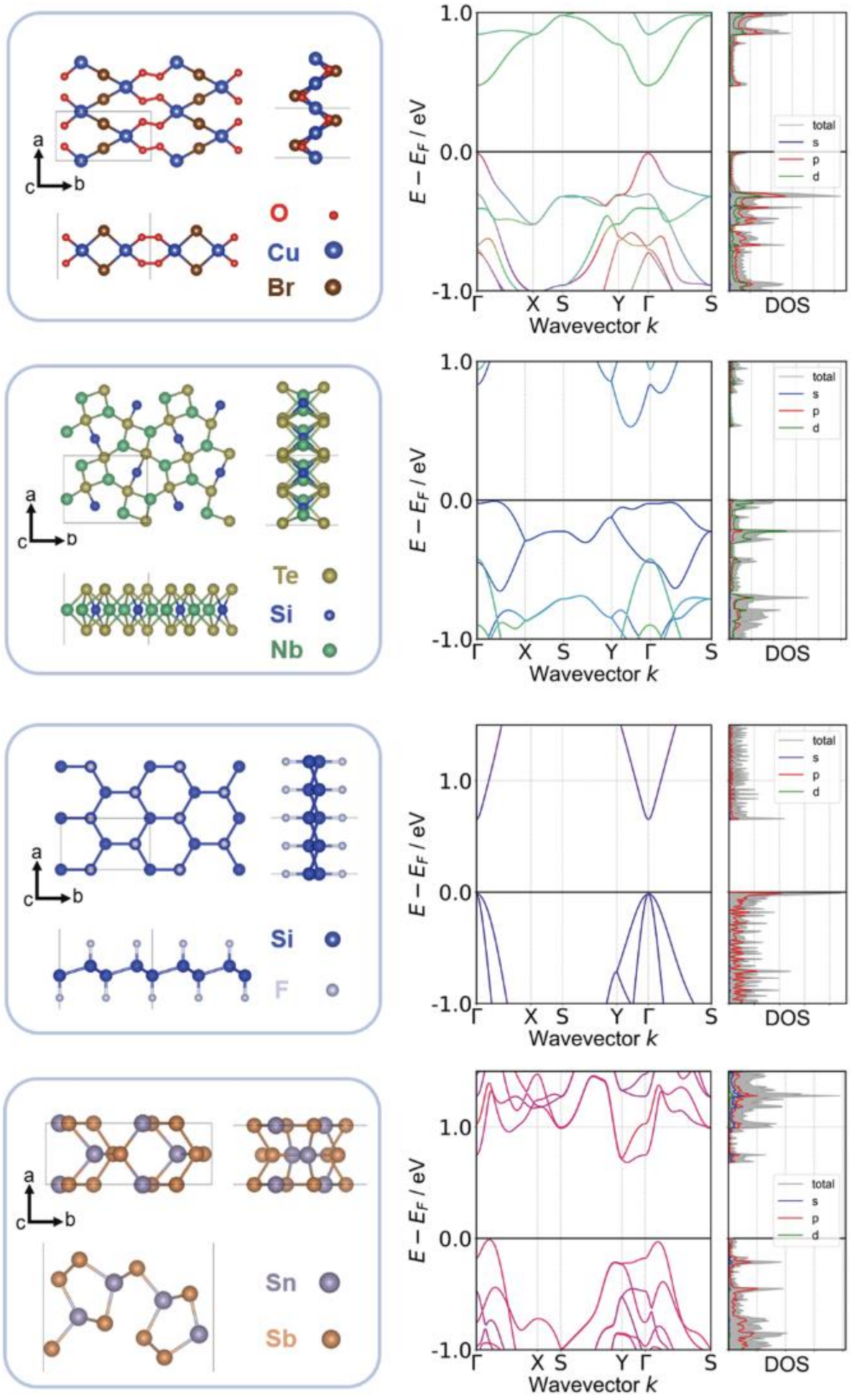

Supplementary Figure 14. Crystal and electronic structures of $CuBrO_2$, Nb_2SiTe_4, SiF and $SnSb_2$.

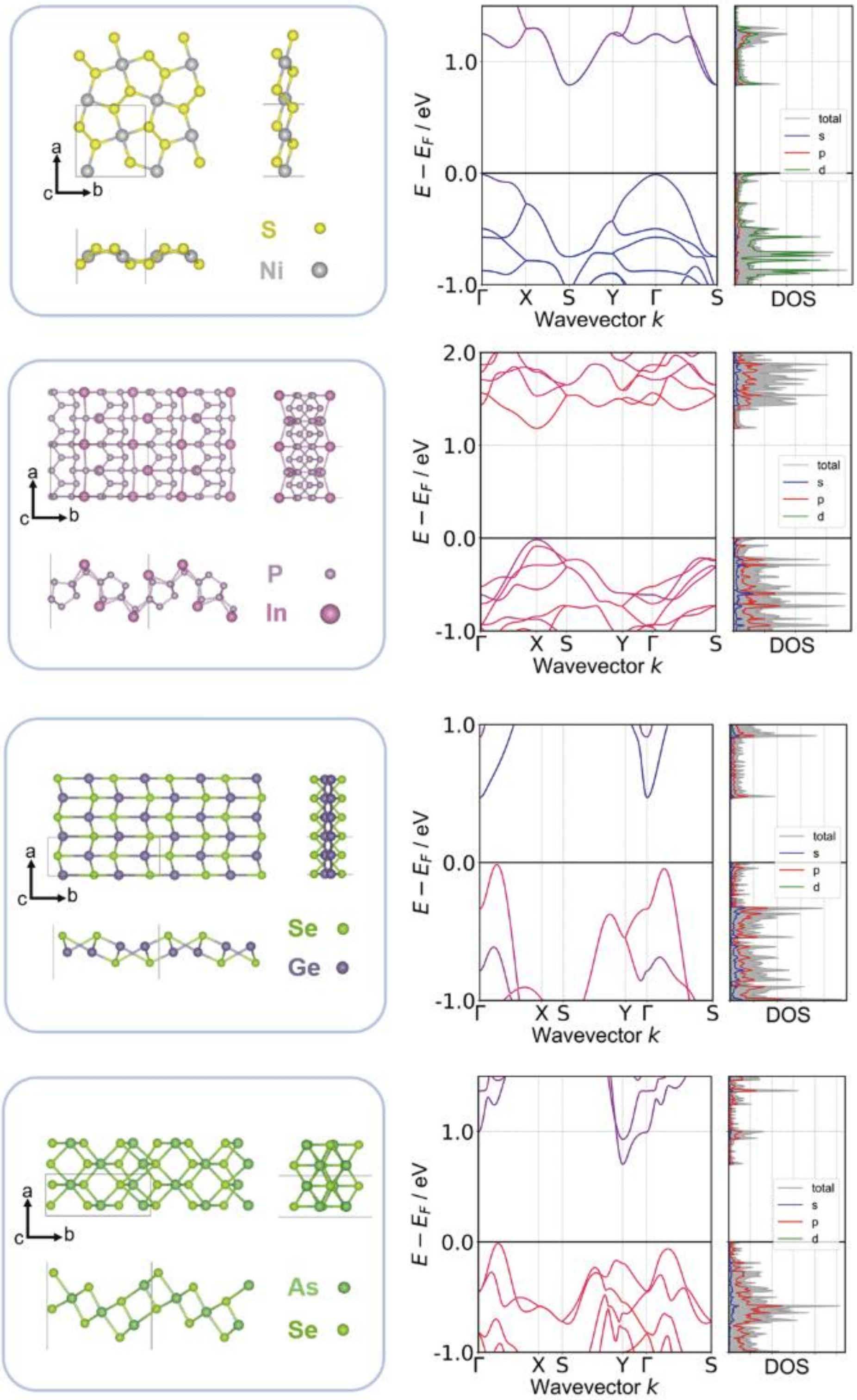

Supplementary Figure 15. Crystal and electronic structures of NiS_2, InP_5, GeSe and As_2Se_3.

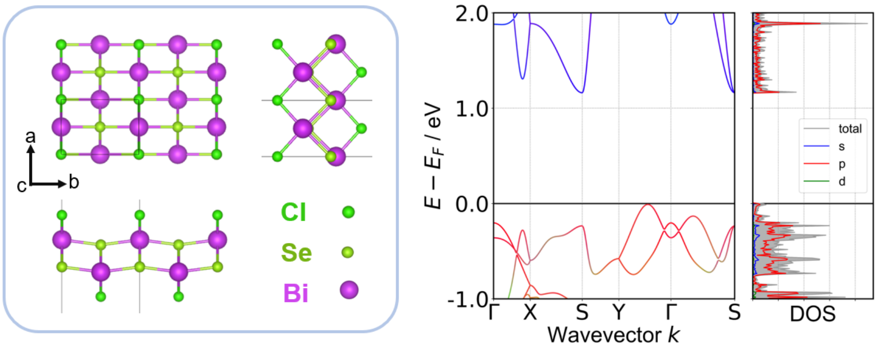

Supplementary Figure 16. Crystal and electronic structures of BiClSe.

Supplementary references:

1 Haastrup, S. *et al.* The Computational 2D Materials Database: high-throughput modeling and discovery of atomically thin crystals. *2d Mater.* **5**, doi:10.1088/2053-1583/aacfc1 (2018).

2 Zhou, J. *et al.* 2DMatPedia, an open computational database of two-dimensional materials from top-down and bottom-up approaches. *Sci. Data* **6**, 86, doi:10.1038/s41597-019-0097-3 (2019).

3 Jha, D. *et al.* ElemNet: Deep Learning the Chemistry of Materials From Only Elemental Composition. *Scientific Reports* **8**, 17593, doi:10.1038/s41598-018-35934-y (2018).

4 Kong, S., Guevarra, D., Gomes, C. P. & Gregoire, J. M. Materials representation and transfer learning for multi-property prediction. *Appl. Phys. Rev.* **8**, 021409, doi:10.1063/5.0047066 (2021).

5 Xie, T. & Grossman, J. C. Crystal Graph Convolutional Neural Networks for an Accurate and Interpretable Prediction of Material Properties. *Phys. Rev. Lett.* **120**, 6, doi:10.1103/PhysRevLett.120.145301 (2018).

6 Lu, S. H. *et al.* Accelerated discovery of stable lead-free hybrid organic-inorganic perovskites via machine learning. *Nat. Commun.* **9**, 8, doi:10.1038/s41467-018-05761-w (2018).
