# Supplementary material for: From bulk effective mass to 2D carrier mobility accurate prediction via adversarial transfer learning
Source: Nat Commun. 2024 Jun 25;15:5391. doi: 10.1038/s41467-024-49686-z (PMC11199574; doi:10.1038/s41467-024-49686-z)
Supplement: Supplementary file 3 — Description of Additional Supplementary Files [file 41467_2024_49686_MOESM3_ESM.pdf]

File Name: Supplementary Data 1
Description: Crystal structure files (POSCAR) of screened high mobility 2D materials.

File Name: Supplementary Data 2
Description: Training data of 2D carrier mobility and bulk effective mass.

File Name: Supplementary Code
Description: Code to perform adversarial transfer learning and predict 2D carrier mobility.
