# Supplementary material for: From bulk effective mass to 2D carrier mobility accurate prediction via adversarial transfer learning
Source: Nat Commun. 2024 Jun 25;15:5391. doi: 10.1038/s41467-024-49686-z (PMC11199574; doi:10.1038/s41467-024-49686-z)
Supplement: Supplementary file 7 — Reporting Summary [file 41467_2024_49686_MOESM7_ESM.pdf]

nature portfolio

Corresponding author(s): Qionghua Zhou, Jinlan Wang

Last updated by author(s): May 8, 2024

Reporting Summary

Nature Portfolio wishes to improve the reproducibility of the work that we publish. This form provides structure for consistency and transparency in reporting. For further information on Nature Portfolio policies, see our Editorial Policies and the Editorial Policy Checklist.

Statistics

For all statistical analyses, confirm that the following items are present in the figure legend, table legend, main text, or Methods section.

n/a	Confirmed	
☐	☒	The exact sample size (*n*) for each experimental group/condition, given as a discrete number and unit of measurement
☒	☐	A statement on whether measurements were taken from distinct samples or whether the same sample was measured repeatedly
☒	☐	The statistical test(s) used AND whether they are one- or two-sided<br>*Only common tests should be described solely by name; describe more complex techniques in the Methods section.*
☒	☐	A description of all covariates tested
☒	☐	A description of any assumptions or corrections, such as tests of normality and adjustment for multiple comparisons
☐	☒	A full description of the statistical parameters including central tendency (e.g. means) or other basic estimates (e.g. regression coefficient) AND variation (e.g. standard deviation) or associated estimates of uncertainty (e.g. confidence intervals)
☒	☐	For null hypothesis testing, the test statistic (e.g. *F*, *t*, *r*) with confidence intervals, effect sizes, degrees of freedom and *P* value noted<br>*Give P values as exact values whenever suitable.*
☒	☐	For Bayesian analysis, information on the choice of priors and Markov chain Monte Carlo settings
☒	☐	For hierarchical and complex designs, identification of the appropriate level for tests and full reporting of outcomes
☒	☐	Estimates of effect sizes (e.g. Cohen's *d*, Pearson's *r*), indicating how they were calculated

Our web collection on statistics for biologists contains articles on many of the points above.

Software and code

Policy information about availability of computer code

Data collection	The data contains three parts, effective mass of bulk materials, carrier mobility of two-dimensional materials and crystal structures of unlabeled two-dimensional materials.<br>The effective mass data for bulk materials has been sourced from the open-source Materials Project database (version 2018), accessible at https://next-gen.materialsproject.org, and DRYAD https://datadryad.org/stash/dataset/doi:10.5061/dryad.gn001.<br>Unlabeled two-dimensional materials have been gathered from two prominent open-source databases: 2Dmatpedia (version 2019) accessible at http://www.2dmatpedia.org, and C2DB (version 2018-12-10) accessible at https://cmr.fysik.dtu.dk/c2db/c2db.html.<br>The carrier mobility data for two-dimensional materials has been compiled from published papers and is organized into two files: DPTmobility.csv and EPCmobility.csv. These datasets are provided via the GitHub repository at https://github.com/XinYu-Chen98/Hybrid-ATL-and-expert-knowledge-for-materials-design.
Data analysis	Density functional theory (DFT) calculations were performed using the Vienna Ab initio Simulation Package (VASP). Additionally, custom algorithms for machine learning were utilized, which are available for access at: https://github.com/XinYu-Chen98/Hybrid-ATL-and-expert-knowledge-for-materials-design

For manuscripts utilizing custom algorithms or software that are central to the research but not yet described in published literature, software must be made available to editors and reviewers. We strongly encourage code deposition in a community repository (e.g. GitHub). See the Nature Portfolio guidelines for submitting code & software for further information.

Data

Policy information about availability of data

All manuscripts must include a data availability statement. This statement should provide the following information, where applicable:

- Accession codes, unique identifiers, or web links for publicly available datasets
- A description of any restrictions on data availability
- For clinical datasets or third party data, please ensure that the statement adheres to our policy

The carrier mobility data generated in this study are provided in the manuscript file, the Supplementary Information files, and Source Data files. Source data are provided with this paper.
The data of 2D materials and bulk effective mass used in this study are available at public websites, C2DB (https://cmr.fysik.dtu.dk/c2db/c2db.html), 2Dmatpedia (http://www.2dmatpedia.org) and MP (https://materialsproject.org/).

Research involving human participants, their data, or biological material

Policy information about studies with human participants or human data. See also policy information about sex, gender (identity/presentation), and sexual orientation and race, ethnicity and racism.

Reporting on sex and gender	No information about sex or gender has been collected in this study.
Reporting on race, ethnicity, or other socially relevant groupings	No information about race, ethnicity, or other socially relevant groupings has been collected in this study.
Population characteristics	No information about population characteristics has been collected in this study.
Recruitment	No participants were recruited in this study.
Ethics oversight	No organization has approved the study protocol.

Note that full information on the approval of the study protocol must also be provided in the manuscript.

Field-specific reporting

Please select the one below that is the best fit for your research. If you are not sure, read the appropriate sections before making your selection.

☐ Life sciences ☐ Behavioural & social sciences ☒ Ecological, evolutionary & environmental sciences

For a reference copy of the document with all sections, see nature.com/documents/nr-reporting-summary-flat.pdf

Ecological, evolutionary & environmental sciences study design

All studies must disclose on these points even when the disclosure is negative.

Study description	This study provides a hybrid framework combines adversarial transfer learning with expert knowledge to predict carrier mobility for 2D materials based on bulk effective mass data. This approach achieves over 90% accuracy in predicting carrier mobilities using only crystal structure data. Leveraging this methodology, we identify 21 new 2D semiconductors with carrier mobilities surpassing silicon, showcasing transfer learning's potential to address material data scarcity. This study is entirely computational, with all material properties evaluated through computational simulations, without any experimental procedures conducted.
Research sample	The research samples encompass both bulk and two-dimensional inorganic materials, reflecting the study's focus on utilizing bulk material data to enhance predictions for two-dimensional materials and identify those with exceptional carrier mobilities. As a computational work, all samples are virtual. Some open-source materials databases are involved, including Materials Project at https://next-gen.materialsproject.org, C2DB at https://cmr.fysik.dtu.dk/c2db/c2db.html and 2Dmatpedia at http://www.2dmatpedia.org.
Sampling strategy	Given the scarcity of available materials data to support machine learning researches, we opted to utilize all accessible materials data without additional sampling or filtering.
Data collection	The materials data are collected by Xinyu Chen from open-access materials databases (Materials Project, C2DB and 2Dmatpedia) and published papers.
Timing and spatial scale	The data for machine learning were initially collected at the outset of this research in December 2021. Validation of the selected materials was subsequently conducted from November 2022 to January 2023, a period necessitated by the computational resources required for carrier mobility evaluation.

Data exclusions	Some materials have been excluded for two reasons. Firstly, duplicates of the same materials sourced from different databases have been eliminated to streamline the dataset; materials with identical space groups and compositions are considered duplicates. Secondly, materials containing radioactive elements have been omitted due to safety concerns in semiconductor applications.
Reproducibility	We have made all the code and data necessary available through a public repository hosted on GitHub. Readers can access it at https://github.com/XinYu-Chen98/Hybrid-ATL-and-expert-knowledge-for-materials-design
Randomization	Our samples were randomized when dividing the data into training and testing datasets, utilizing the train-test-split function built into scikit-learn, which is a common practice in the field of machine learning.
Blinding	Our study, which focuses on predicting material properties, does not require blinding. This is because our research deals with the physical and chemical properties of materials, rather than subjective evaluations or observations.

Did the study involve field work? ☐ Yes ☒ No

Reporting for specific materials, systems and methods

We require information from authors about some types of materials, experimental systems and methods used in many studies. Here, indicate whether each material, system or method listed is relevant to your study. If you are not sure if a list item applies to your research, read the appropriate section before selecting a response.

Materials & experimental systems

n/a	Involved in the study
☒	☐ Antibodies
☒	☐ Eukaryotic cell lines
☒	☐ Palaeontology and archaeology
☒	☐ Animals and other organisms
☒	☐ Clinical data
☒	☐ Dual use research of concern
☒	☐ Plants

Methods

n/a	Involved in the study
☒	☐ ChIP-seq
☒	☐ Flow cytometry
☒	☐ MRI-based neuroimaging

Plants

Seed stocks	No seed is involved in this study.
Novel plant genotypes	No plant is involved in this study.
Authentication	No plant is involved in this study.
